# Efficient content caching for 5G assisted vehicular networks

**DOI:** 10.1038/s41598-024-54486-y

**Published:** 2024-02-18

**Authors:** Faareh Ahmed, Badr Alsamani, Mohammed Alkhathami, Deafallah Alsadie, Norah Alosaimi, Badriya Alenzi, Lewis Nkenyereye

**Affiliations:** 1 School of Electrical Engineering & Computer Science (SEECS), National University of Sciences & Technology, Islamabad, Pakistan; 2 Information Systems Department, College of Computer and Information Sciences, Imam Mohammad Ibn Saud Islamic University (IMSIU), Riyadh, 11432 Saudi Arabia; 3 Information Systems Department, Umm Al-Qura University, Mecca, Saudi Arabia; 4 Department of Computer and Information Security, Sejong University, Seoul, 05006 South Korea

**Keywords:** Content caching, Fog nodes, Smart cities, Computer science, Information technology

## Abstract

Traffic congestion is one of the major challenges faced by daily commuters in smart cities. An autonomous transportation system with a 5 G-based Cellular Vehicle-to-Everything (C-V2X) communication system is the solution to meet the traffic challenges faced in smart cities. Vehicular networks provide wireless connectivity to enable a large number of connected vehicle applications. Vehicular networks allow vehicles to share their emergency and infotainment traffic by following vehicle-to-vehicle (V2V) or by using vehicle-to-infrastructure (V2I) communication. The infrastructure of vehicular networks mainly comprises multiple Road Side Units (RSUs). Fog computing nodes are placed adjacent to these RSUs to provide quick access to vehicles. For infotainment traffic, vehicles intend to download their required content from the content provider. Caching the same contents from the nearby fog computing node significantly reduces delay with improved quality of service. As there are millions of contents with varying sizes, caching all demanded contents on these fog nodes is not possible due to their limited caching capacity. In this work, we propose an improved content caching scheme for fog nodes to satisfy vehicles and content providers for fair content placement. The proposed algorithm is based on a modified Gale–Shapley technique that considers factors such as content popularity, vehicle connectivity, and quality of the communication channel to optimize the content caching process. Simulation results show that the proposed technique caches a higher number of popular contents with lower downloading time.

## Introduction

Smart cities are developing rapidly because they overcome the challenges faced in growing urban cities and provide ease in human lifestyles, such as environment-friendly pollution-free atmosphere, improved public safety, reduced traffic congestion with entertaining drive, better waste management, and better resource management^1–3^. Smart cities provide sustainable development with increased economic growth by empowering citizens’ engagement. Advancements in technology such as ubiquitously connected IoTs and deployment of 5G and 6G communications augment the smart cities applications^4,5^.

Road accidents with poor traffic management are one of the major challenges faced in smart cities. A safe and entertaining drive is one of the major requirements for vehicle passengers^6,7^. Vehicular networks are new emerging networks that are developing due to the advancement in wireless communication and automotive technologies and not only meet the challenges faced in road congestion but also provide entertainment to passengers to watch their favorite content during their journey. Vehicular networks are created when mobile vehicles on the road installed with wireless equipment (homogeneous or heterogeneous) come into contact with other vehicles. Networks formed from these interactions are identified as Vehicular Ad-hoc Networks (VANETs), which are described as the communication between vehicles or with vehicles and nearby placed static equipment, typically defined as Road Side Units (RSUs). Three type of communication is available in a vehicular network, that is Vehicle to Vehicle (V2V) communication, Vehicle-to-infrastructure (V2I), Vehicle-to-everything (V2X)^8–10^. Out of these, V2V communication is mostly used for safety application, and V2I and V2X communication is mostly used for traffic management and infotainment

Vehicles installed with wireless transceivers called On Board Units (OBUs) connect with the RSUs during their journey. RSUs have the storage capacity where they cache the required data for the vehicles on the road^11,12^. RSUs are either installed by Traffic Control Centers (TCC) or the automobile manufacturers for the support of vehicles on the road^13^.

TCC is located at a central location in the city and contains different servers that receive data from city-wide RSUs. TCC with the help of cooperative navigation and speed management systems, offers traffic management applications to provide smooth traffic movement by providing faster routes, supplying adaptive traffic signals, and giving real-time traffic information of the locality, such as accidents, under construction road sections and steep road segments [13]. In addition to traffic management services, TCC also provides infotainment services to passengers that include the location of the nearby hotels, weather forecast, music, and live streaming services, so that the requested information can be provided to the commuter at the right moment^14,15^.

With built-in touchscreens and mobile user operating systems, like Android Auto and Apple CarPlay, it can be anticipated that shortly build-in vehicular broadband communication and vehicular infotainment of high-quality applications are going to be exceedingly demanding^16^. Therefore, to overcome the ever-increasing demand in vehicular networks for contents from numerous applications and infotainment services, it is necessary to cache the popular contents nearer to the end user to reduce the both traffic load of the network and data delivery delay. This demands a faster downloading data rate with better network connectivity, which is one of the major constraints in vehicular networks because fetching the required information from the remotely placed servers causes delay. To overcome this delay, the demanding resources need to be placed in close vicinity of these vehicles such as fog computing nodes.

Fog computing covers essential services from the network center to the network edge, that can efficiently resolve the problems of bandwidth, low latency, and vulnerability instigated by the prolonged distances^17^. Fog computing nodes are encouraged over cloud computing due to their placement in close vicinity of accessing nodes^18^. Fog computing is a supplement to cloud computing and is preferred due to the following two reasons:

1. They provide fast and easy access to nodes due to their placement in close vicinity.
2. The vulnerability of wireless to access wireless network is significantly reduced by limiting the network access.

In a vehicular network, an RSU with caching capacity makes it a fog computing node. Fog computing nodes provide execution of offloaded tasks as well as provide the caching capacity for the placement of demanding contents resulting in fast access of the demanding contents. The fog node has limited caching capacity and it is not possible to cache all the requested contents on it. It is therefore required to cache the most demanding contents on the fog node to fulfill the requirements of the majority of the vehicles.

In this work, an efficient content caching mechanism is proposed to cache the most demanding contents in such a way that the majority of the vehicles fetch their demanded contents at a faster data rate. The salient features of the proposed scheme are:

- To evaluate the utility function of RSUs and request contents to prepare the preference lists. The utility function of each RSU is based on content popularity, remaining connecting time of vehicles, and quality of communication channel. Similarly, the utility function of each content is determined by calculating the cost of placing content on each RSU.
- Modify the Gale–Shapley algorithm from one-to-one matching to one-to-many matching for content caching to satisfy the requested content demand of the majority of the vehicles.

The rest of the paper is organized as: Section “Related work” is about the recent research work in content caching vehicular networks. The system model and proposed scheme are discussed in Sections “System model” and “Proposed scheme” respectively. Simulation and results with comparative analysis are described in Section “Results and analysis” and Section “Conclusion” concludes the manuscript.

## Related work

There have been several proposals that improve the caching process in vehicular and mobile networks. Most of the work focuses on efficient content placement according to the popularity of the content.

In^19^, the work focuses on efficient content caching in a Unmanned Aerial Vehicle (UAV) network. The UAVs cache popular content and act as service providers to the ground stations. A key feature of the proposed technique is that it considers the popularity of content according to geographic areas within a particular time interval. The work improves access to content and reduces the overall delay in retrieving content.

The technique proposed by^20^ uses an Artificial Intelligence (AI) assisted technique to improve caching in the vehicular network. A deep reinforcement learning technique is used by the authors and an optimal policy function is learned by the developed algorithm. The transmission delay and cache hit ratio are improved by the proposed technique.

The work in^21^ considers a satellite-assisted UAV and vehicular network. The goal is to improve the delay of content retrieved from the backbone network and also reduce energy consumption. The work uses an efficient coding technique for caching and achieves improved content retrieval at a low energy cost.

In^22^, a prediction technique is proposed by the authors to improve the caching in vehicular networks. The goal of the algorithm is to predict vehicle mobility and utilize it to store the contents at the RSUs. Vehicles are further divided into clusters to manage the content caching. Simulation results show that the proposed technique improves the content retrieval time.

The work in^23^ also utilizes the idea of clustering. The goal is to select the cluster head vehicle in each cluster and allocate the cached content to it. A prediction scheme is also employed to forecast the popularity of content. Moreover, the base station is also used to cache popular content, however, the load on it is reduced by using cluster heads. The download delay of content is reduced by the proposed technique.

As compared to the previous work, our work considers several caching related factors such as content popularity, V2I connectivity, V2I channel quality and preference of both the vehicles and the RSUs. Furthermore, we also develop a one-to-many matching algorithm to map vehicle contents to the storage space on the RSUs.

## System model

**Figure 1:**
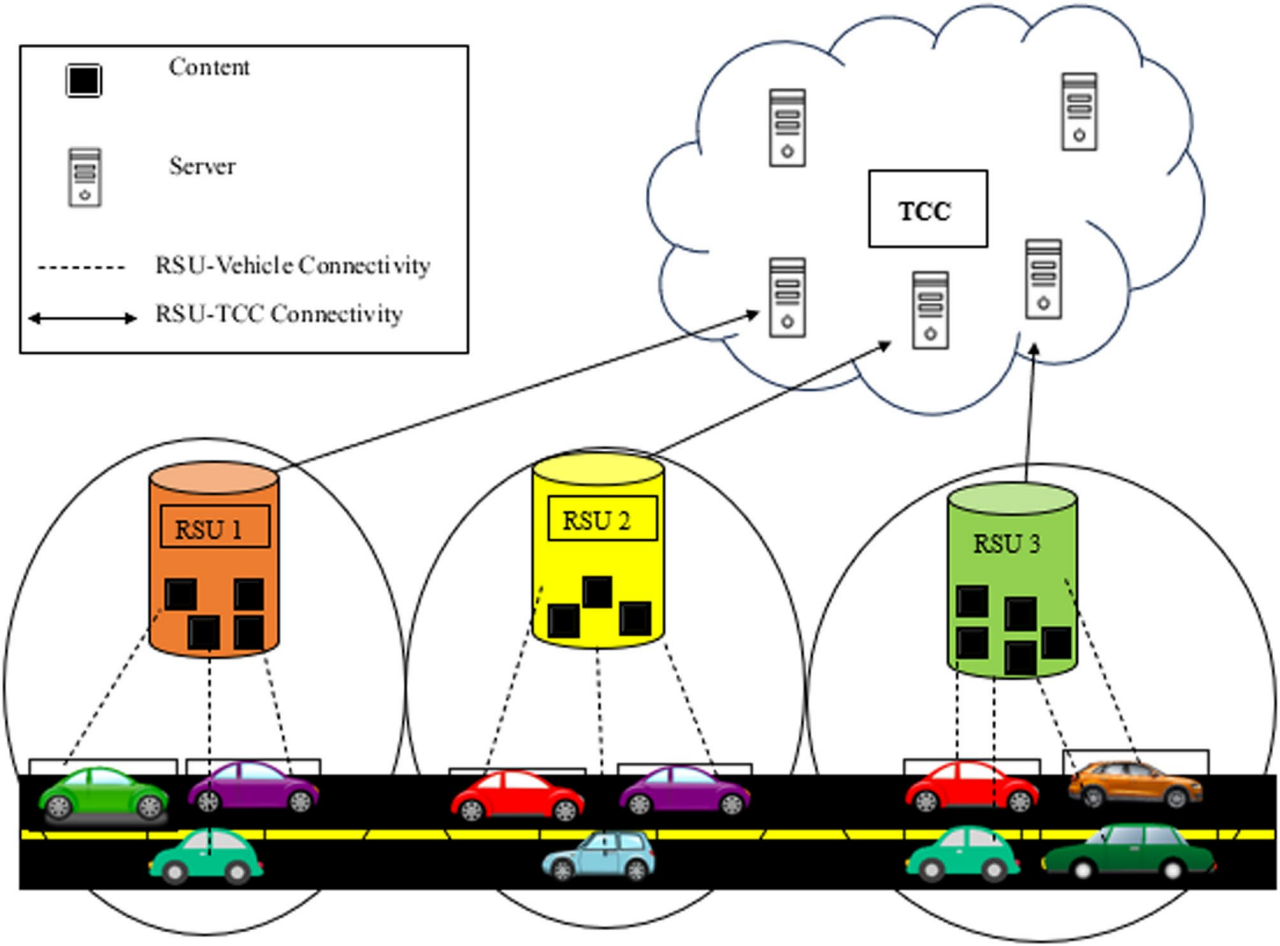
System model.

Suppose there are *R* number of RSUs and *V* vehicles pass through these RSUs in time *t* as shown in Fig. 1. The transmission power of all RSUs is the same while their coverage area differs due to the change in geographical environment. The total number of content ($C_{req}$) to be originated by *V* vehicles and the sizes of these contents are represented by $CS_{req}$. As $x^{th}$ content of the $y^{th}$ vehicle is represented by $C_{x,y}$, the size of this particular content will correspond to $CS_{x,y}$. If *v* vehicles requested for *M* number of contents then $C_{req}$ and $CS_{req}$ for a specific time *t* is calculated as:

1 $$\begin{aligned} C_{req}= & {} \sum _{x=1}^{M}\sum _{y=1}^{V} C_{xy} \end{aligned}$$

2 $$\begin{aligned} CS_{req}= & {} \sum _{x=1}^{M}\sum _{y=1}^{V} CS_{xy} \end{aligned}$$

All the fog nodes have the same caching capacity $C_{Cap}$ and fog nodes can only cache contents within their caching limit. If $i^{th}$ content ($C_i$) with size $CS_{i}$ is selected to be cached on one of the fog nodes and there are *R* number of fog nodes with accumulated caching capacity of $Fog_{cache}$ then total number of contents $C_{Alloc}$ along with their sizes $CS_{Alloc}$ allocated on all fog nodes are calculated as:

3 $$\begin{aligned} C_{Alloc}= & {} \sum _{i=1}^{R} C_{i} \end{aligned}$$

4 $$\begin{aligned} CS_{Alloc}= & {} \sum _{i=1}^{R} CS_{i}... | CS_{Alloc} \epsilon CS_{req} \& \& CS_{Alloc}\le Fog_{cache} \end{aligned}$$

All the vehicles in vehicular networks are in direct connection with one of the *R* RSUs throughout their journey. The connectivity time of a vehicle is connected with its speed as well as the coverage area of the RSU. If velocity of $n^{th}$ vehicle is $v_n$ and coverage area of $n^{th}$ RSU is $\phi _n$, then connectivity time of the vehicle $t_n$ is calculated as:

5 $$\begin{aligned} t_{n} = \phi _n \times v_n \end{aligned}$$

If *m* number of vehicles pass through the RSU $\phi _n$ simultaneously, then the average time spent by a vehicle in the coverage area of that RSU ($t_{ave}$) is calculated as:

6 $$\begin{aligned} t_{ave} = \frac{\sum _{n=1}^{m}(\phi _n \times v_n)}{m} \end{aligned}$$

and the average velocity of a vehicle ($v_{ave}$) is calculated as:

7 $$\begin{aligned} v_{ave} = \frac{\sum _{n=1}^{m}(v_n)}{m} \end{aligned}$$

The downloading time of content by a vehicle primarily depends upon the data rate, and size of the content. The data rate (DR1) to download the contents by vehicle from remotely placed servers is calculated as:

8 $$\begin{aligned} DR1 = log_2 (1 + \Psi _{S,V}) \end{aligned}$$

where $\Psi _{S, V}$ is the Signal Noise Ratio (SNR) between server and vehicle v. Similarly, the data rate (DR2) to download contents by vehicle from the cache of the fog node is calculated as:

9 $$\begin{aligned} DR2 = log_2 (1 + \Psi _{F,V}) \end{aligned}$$

where $\Psi _{F, V}$ is the Signal-to-Noise Ratio (SNR) between the Fog node and vehicle v.

The downloading time *DT*1 and *DT*2 of content having size *CS* from the remote server and fog nodes respectively are calculated as:

10 $$\begin{aligned} DT1 = CS/DR1 \end{aligned}$$

11 $$\begin{aligned} DT2 = CS/DR2 \end{aligned}$$

## Proposed scheme

Vehicles connected in vehicular networks intend to download their required contents without compromising the delay. Downloading their demanded contents from the remote servers may cause some unacceptable delay. That’s why, they are preferred to be cached on nearby RSUs acting as a fog node. Most of the content providers are willing to place their contents on fog nodes even though, there is a caching price for each fog node. However, all fog nodes can’t cache all the contents due to limited caching capacity. This work proposes an efficient content caching policy for TCC to cache the contents on fog caching nodes to facilitate a maximum number of vehicles. The proposed scheme comprises two parts.

1. Utility function is calculated for both fog nodes and content providers, which helps content providers in determining their preference list.
2. The content caching problem is solved by applying a modified Gale–Shapley stable matching problem.

### Utility function calculation

Users in vehicles and content providers intend to cached their required contents on fog nodes for fast downloading. The fog nodes in vehicular networks can not fulfill their complete requirements. To fulfill the maximum of their demands, the Gale–Shapley stable matching algorithm is proposed that is based on the preference choices of content providers and fog nodes. In this work, the preference list of both content providers to cache their contents of fog node and the preference list of fog node to cache the contents is based on cost value determined in the following sections.

#### Utility function for content providers

The utility function of content providers for caching their content on one of the RSUs is based on the cost, that the content provider has to bear in placing its content on each RSU. The cost is calculated by considering the revenue generated by these nodes and the caching price to place its contents on each of the available RSUs. If the caching price of fog node $R_i$ in placing content of size $S_a$ for a specified time period is $P_a$ and the expected revenue generated from each content retrieval of content by vehicle is $R_a$ and there are *m* number of requests expected to access the content *a* then utility function of content *a* in caching on $R_i$ fog node ($Util^a_{Ri}$) is calculated as:

$$\begin{aligned} Util^a_{Ri} = \sum _{a=1}^{m} R_a - P_{sa} \end{aligned}$$

Similarly, the cost of content *a* to cache on each fog node is computed. The smaller the cost calculated for a fog node, the higher will be its preference.

#### Utility function for fog nodes

Utility functions for fog nodes to prepare a preference list of contents to be cached on fog nodes are based on the following parameter values.

- Relative popularity of each content in an RSU range ($\psi _{C, F}$, and it is calculated as the ratio between content requests received and the total number of contents received by fog node. If there are *k* requests received for *C* content in a fog coverage area of *F*, and total requests for all content are $C_{max}$, then ($\psi _{C,F}$ for content *C* is calculated as:
  $$\begin{aligned} \psi _{C,F} = \frac{\sum _{c=1}^{k} C_{c}}{C_{max}} \end{aligned}$$
- Remaining connectivity time ($T_{rem(C, F)}$) of content requesting vehicles and it is calculated as:
  $$\begin{aligned} T_{rem(C,F)} = \frac{\sum _{c=1}^{k} S_{c,F}}{V_{c}} \end{aligned}$$
  here, $S_{C,F}$ is the remaining distance of *c* content requesting vehicle with *F* RSU, and $V_{c}$ is the speed of *c* content requesting vehicle.
- Average Signal to Noise ratio ($\gamma _{F, V}$) between vehicle and RSU, that is based on the signal strength received by a vehicle throughout its connectivity with the same RSU.

The utility function ($Util^{Ri}_C$) for RSU $R_i$ for *m* number of requests received for content *C* is calculated as:

$$\begin{aligned} Util^{Ri}_C = \frac{1}{\psi _{C,F} \times T_{rem(C,F)} \times S_{C,F}} \end{aligned}$$

The lower the computed value, the higher will be the preference of the content.

### Content selection criteria

Due to the high ratio between requested contents and the caching capacity of fog nodes, it is not possible to entertain all requested contents to be cached on fog nodes. The rationale is to cache the most popular content on the fog node to meet most of the requests. In this work, a modified stable matching Gale–Shapley algorithm is proposed, that allows multiple contents to be cached on a fog node.

**Figure 2:**
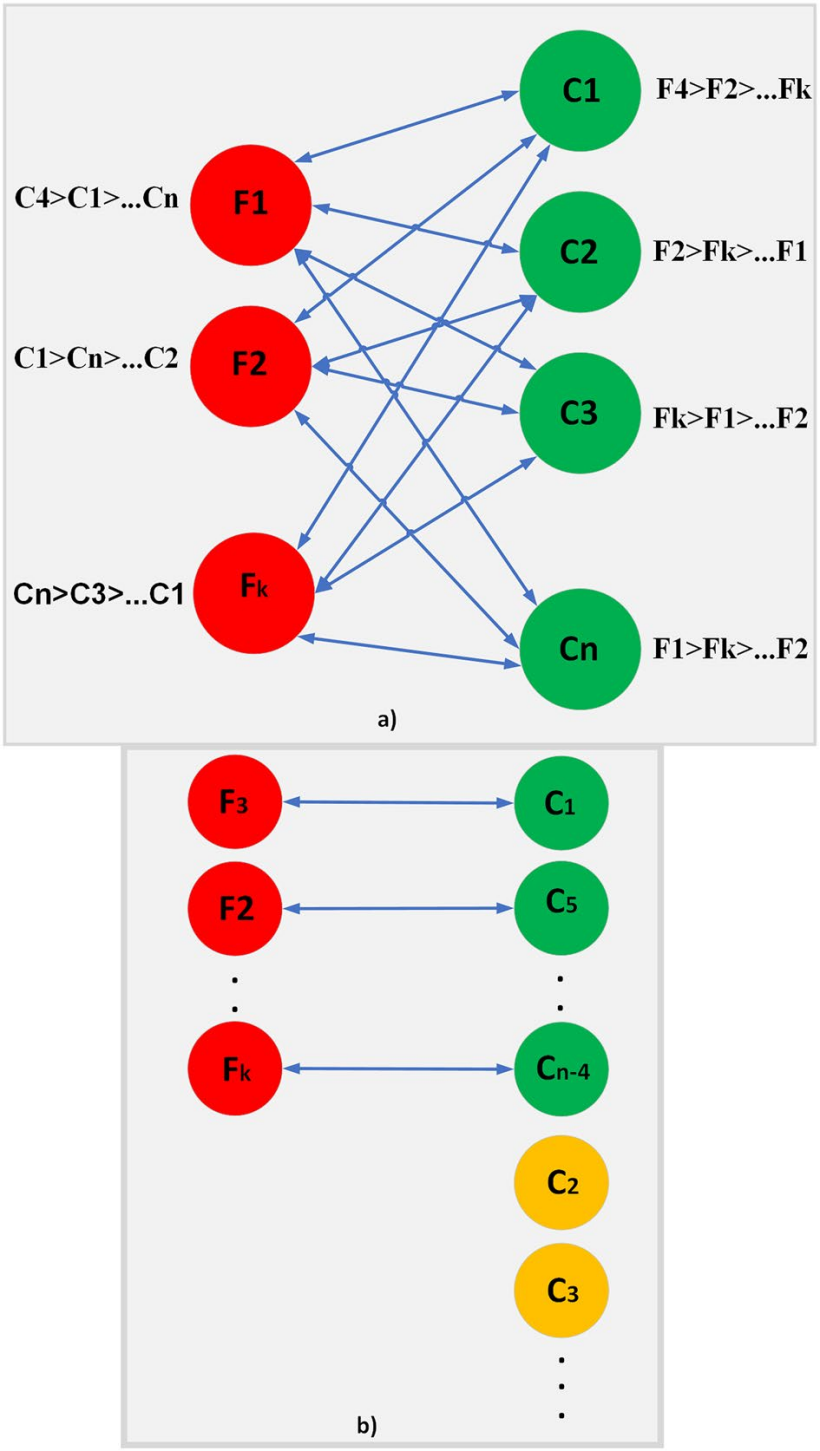
A Bipartite graph representing the working of stable matching algorithm (**a**) List of contents and Fog nodes with their preferences (**b**) Placement of each content on caching fog node along with unmatched contents.

A Gale–Shapley algorithm provides stable matching and is represented with a bipartite graph as shown in Fig. 2. The TCC after receiving all the content requests from the vehicles assigns a preference list of fog nodes. Similarly, it prepares content preferences for caching its content on a fog caching node. It can be seen that each of the represented fog nodes $F_1$, $F_2$,...$F_k$ has a priority list of contents to be cached on it as described in section “Utility Function Calculation”. Similarly, $C_1$, $C_2$, $C_3$,...$C_n$ are contents that are interested in being placed on a fog caching node with their calculated preference list as described in section “Utility Function Calculation”. We consider the size of these contents ($S_n$) to be quite high as compared to the caching capacity of these fog nodes, such as $S_n>>k$. TCC after applying the algorithm, matches and assigns each fog node a separate content from its preference list, and the remaining contents remain there as mentioned with yellow in Fig. 2b.

Initially, all the fog nodes are supposed to have the same caching capacity. After the first iteration, the caching sizes of all fog nodes are not uniform. The allocated contents are removed from the already prepared preference list. Similarly, those contents, whose sizes are greater than the available caching capacity of the fog node are also removed from the preference list of each fog node. The process continues, once the preference list of fog nodes is empty. The complete content caching process is shown in Algorithm 1.

**Algorithm 1:**
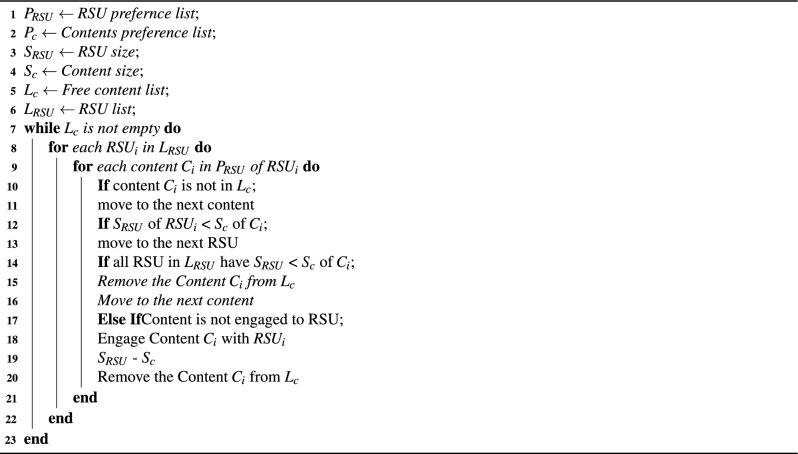
Content caching criteria.

## Performance analysis

To evaluate the performance of the proposed scheme, a simulation environment in the light of the system model is created. The comparative performance is analyzed with a one-to-one stable matching algorithm and randomly deployed requesting contents.

This section is divided into two subsections. The simulation setup and detail of parameters are described in subsection “Simulation Parameters” and the comparative performance of the simulated results are described in subsection “Results And Analysis”.

### Simulation parameters

To compare the performance of the proposed scheme with the other two schemes, a simulation environment is created in MATLAB. In this simulation scenario, the number of RSUs with caching capacity fog nodes ranging from 2 to 5 with randomly chosen coverage area ranges from 2 to 3 km. The caching capacity of these fog nodes ranges from 2 to 4 GB with an increment of 1. There are randomly deployed vehicles in different locations of the RSUs coverage area requesting 60 contents.

Vehicles are moving with different speed ranges between 25 and 45m/s. The contents that are not cached on fog nodes are fetched from servers placed in the TCC location and the data rate for the direct vehicle to TCC communication is taken as 4 MBytes/s. For vehicle-to-RSU communication, the data rate is taken as 20 MBytes/s^24^.

A complete list of simulation parameters is shown in Table 1. Monte Carlo-based simulations are performed for a fair performance evaluation of the proposed scheme with other schemes and results are obtained as an average of $10^5$ experiments.

**Table 1:** Simulation parameters.

Parameter	Value
Number of RSUs (R)	2$\sim$5
Number of vehicles within an RSU coverage range (N)	40$\sim$80
RSU coverage range	10,000 m
Content Size Range (MB)	200$\sim$500
Vehicle speed (m/s)	25$\sim$45
Data rate for vehicle to TCC link (MBytes/s)	4
Data rate for vehicle to RSU link (MBytes/s)	20
RSU Cache size (MBytes)	400$\sim$1000
Content size (MBytes)	20$\sim$40

### Results and analysis

The performance of our proposed scheme is evaluated in terms of the percentage of contents cached on RSUs, accumulated and average content downloading time, and downloaded data. The simulation results are taken for varying numbers of RSUs as well as their varying content caching capacity and varying number of content requests received.

#### Popularity of cached contents

The popularity of cached contents on RSUs is calculated by finding the percentage of the total number of contents cached on all fog nodes against the total number of requested contents received by TCC.

**Figure 3:**
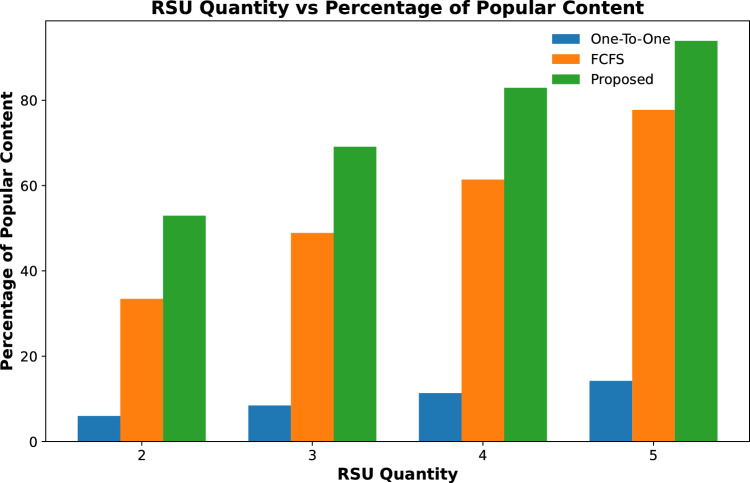
Percentage of requested contents cached against varying quantity of RSUs.

**Figure 4:**
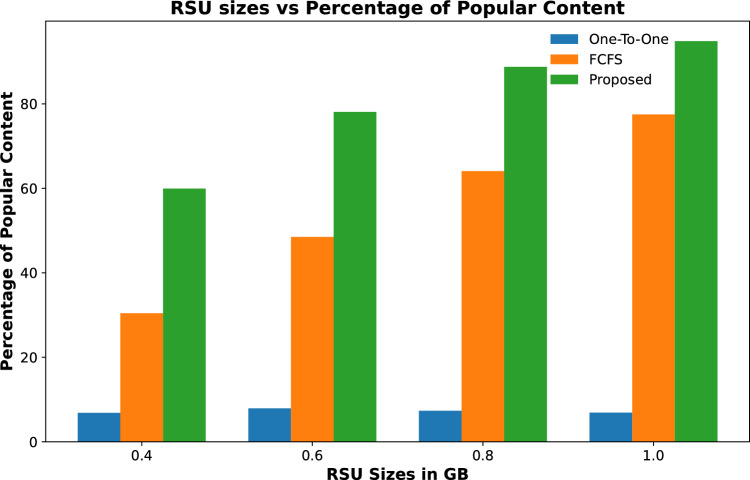
Percentage of requested contents cached against varying caching capacity of RSUs.

**Figure 5:**
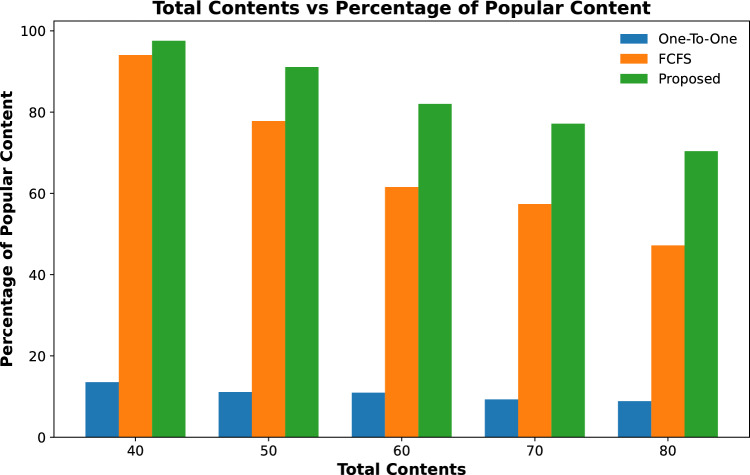
Percentage of requested contents cached against varying contents requests.

Results shown in Fig. 3 represent the percentage of total contents that are cached on fog-enabled RSUs for an increasing number of RSUs. The results show that the proposed scheme cached 25% of requested contents when the number of RSUs is only 2 and it increases to about 38% for 5 RSUs with the same caching capacity. However, the contents cached on the other two schemes are far less than the proposed for all varying numbers of RSUs. The same trend is shown in Fig. 4 when the content caching capacity of 3 fog-enabled RSUs is increased from 2 to 4 GB of data. It is evident from the results, that number of cache contents increased with the increase in caching capacity of RSUs from 42 to 67%. However, the content caching capacity of one-to-one and FCFS is far less than the proposed scheme.

Figure 5 shows the percentage of popular contents cached on RSUs for varying numbers of content requests. The results show that when content requests are less than maximum the contents are cached on fog-enabled RSUs. However, when the number of content requests increases then the percentage of cached contents on RSUs decreases gradually. It is evident from these results that the proposed scheme still manages to cache a higher percentage of popular content on RSUs as compared to the other two schemes.

#### Contents downloading time

The content downloading time of a content requesting vehicle is calculated as, when it transmitted the content request and the time of whole content is received by it. In this work, we have considered three possible scenarios of vehicles fetching their required content.

1. If a vehicle has to fetch its content from the remotely placed server, then it fetches this information with a lower data rate (*DR*1) with a higher downloading time as mentioned in Eq. (10).
2. If the requested content of a vehicle is placed on the attached fog node-based RSU then the requested content will be downloaded in a significantly smaller time as both the vehicle and the RSU are on the same network and offer a high data rate (*DR*2). The downloading time from the attached fog node is calculated as mentioned in Eq. (11).
3. If a vehicle cannot download the whole of its content from the fog node due to shorter connectivity time with the fog node then it has to fetch some of the data from a remotely placed server. Suppose a node has to fetch *CS* content, and a part of the content ($CS_1$) is downloaded from the connected fog node and the rest of the content from the remotely placed server then total downloading time (*DT*) is calculated as:
  $$\begin{aligned} DT = \frac{CS_1}{DR1} + \frac{CS-CS_1}{DR2} \end{aligned}$$

**Figure 6:**
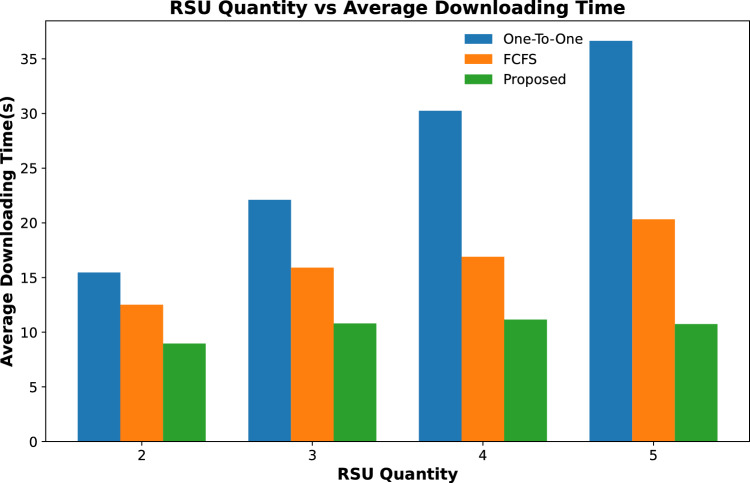
Average time to download content against varying quantity of RSUs.

**Figure 7:**
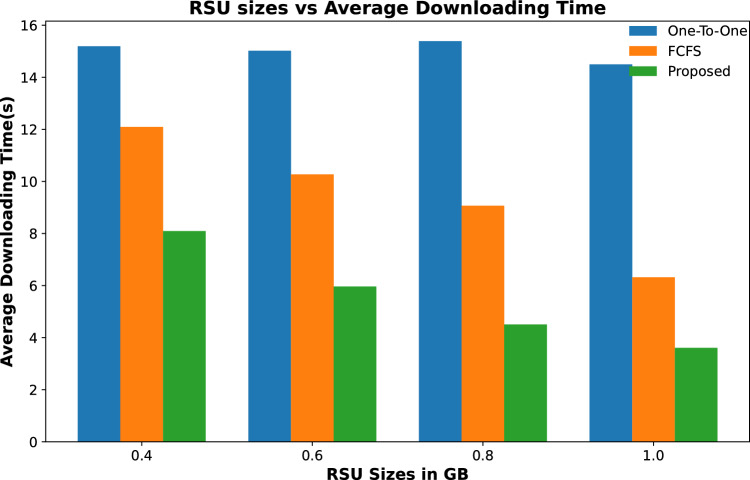
Average time to download content against varying cache size of RSUs.

Results are shown in Figs. 6 and 7 calculate the average time to download a fixed number of contents by all requesting vehicles throughout their journey. Figure 6 shows that the average downloading time to download all requesting contents for varying numbers of RSUs is less than the other two schemes. The downloading time keeps on decreasing with the increased number of RSUs. This is due to the high number of contents that are cached on these RSUs and most of the requested contents are downloaded with a high data rate and are downloaded in less amount of time. The same trend is followed in Fig. 7, where the average downloading time of the same number of requesting nodes is calculated for varying caching sizes of fog-enabled RSUs. It is evident from the results that the proposed scheme downloads the requested content in relatively less amount of time as compared to the other two schemes. In addition, the average downloading time decreases with the increase in caching capacity of fog nodes.

**Figure 8:**
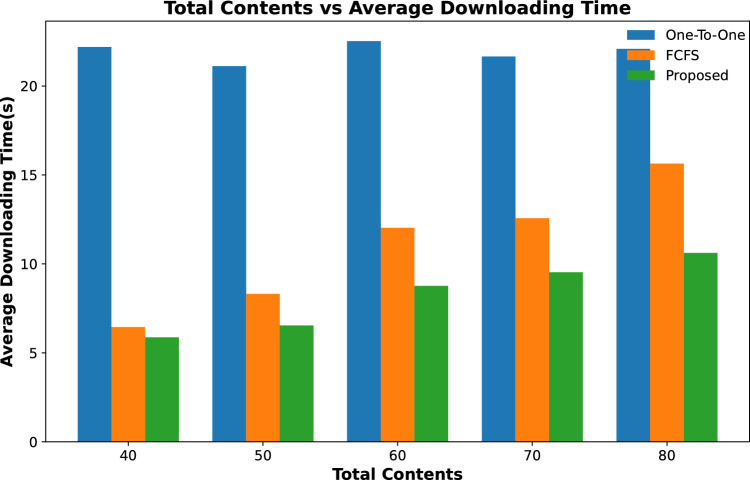
Average time to download content against varying number of content requests.

Figure 8 shows the average downloading time of contents from a varying number of content requests from vehicles when the number of RSUs along with their caching capacity remains unchanged. The results show that the average downloading time to fetch all requested content increases with the increase in content requests for all three schemes. However, in the proposed scheme, this downloading time is significantly smaller than in the other two schemes.

**Figure 9:**
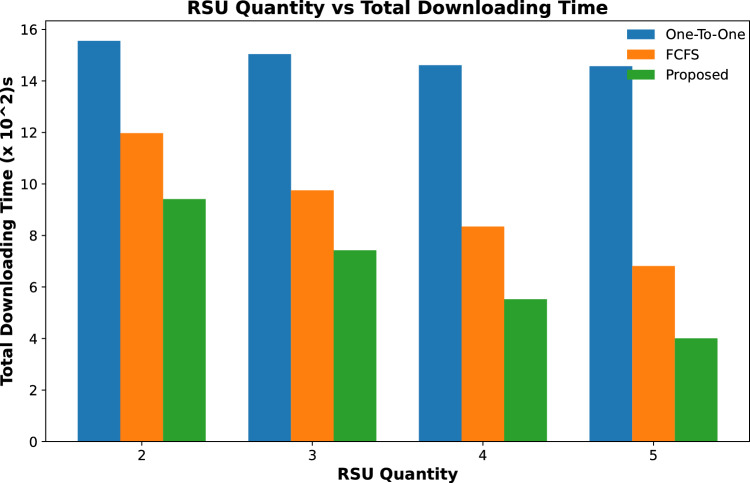
Accumulated time to download all contents against varying number of RSUs.

**Figure 10:**
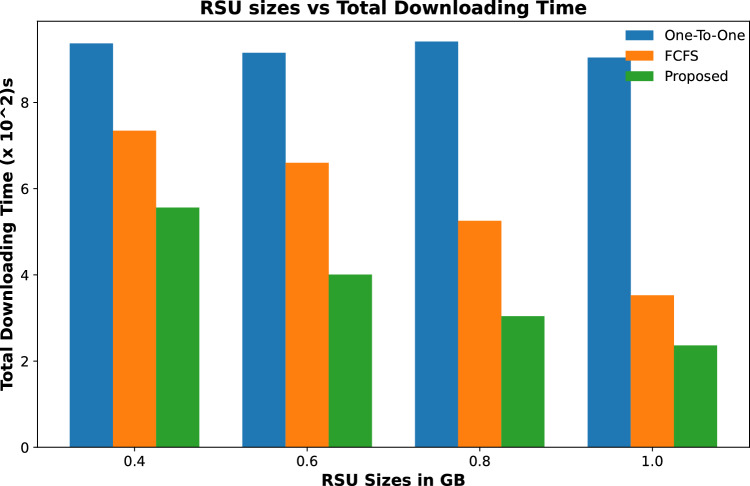
Accumulated time to download all contents against varying caching capacity of RSUs.

Figures 9 and 10 show the total time calculated in downloading all requested contents by vehicles for varying numbers of RSUs and for varying caching capacity respectively, while the number of contents requests is fixed. The content downloading time keeps on decreasing with an increase in the number of RSUs as it caches most of the requested contents resulting in a decrease in downloading time as shown in Fig. 9. The same trend follows for varying content caching capacity of RSUs for the same number of requested contents with a fixed number of RSUs and their caching capacity as shown in Fig. 10. It is evident from these results that the downloading time of all requested contents in our proposed scheme is significantly less than the other two schemes.

**Figure 11:**
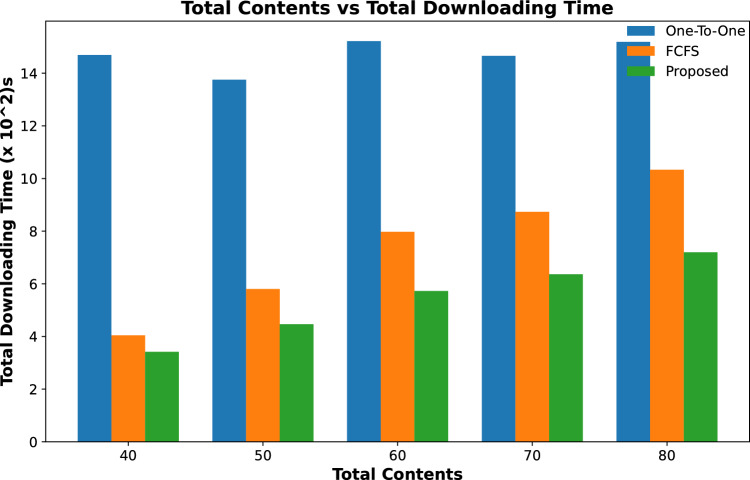
Accumulated time to download all contents against varying number of contents requests.

Results shown in Fig. 11 calculate the total time required to download all requested contents from a cached and remote server. It is evident from the results that the total downloading time of the proposed scheme is less than the other two schemes. However, this time keeps on increasing with the increasing number of content requests time in a specific time period.

#### Downloaded data

**Figure 12:**
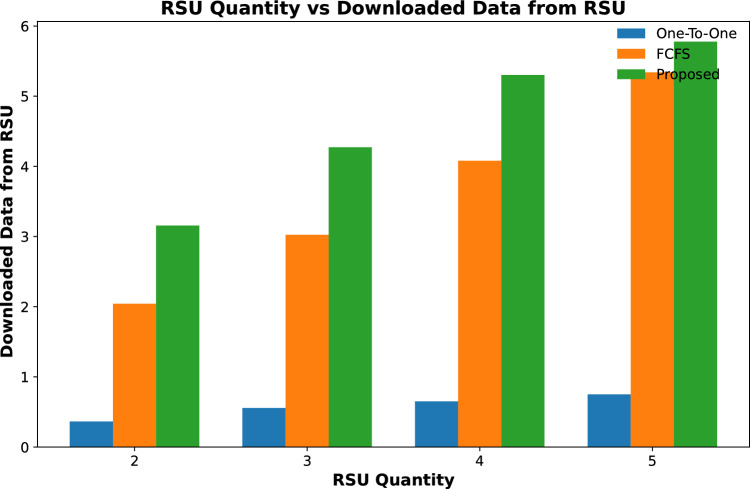
Data downloaded from contents cached on RSU against varying numbers of RSUs.

**Figure 13:**
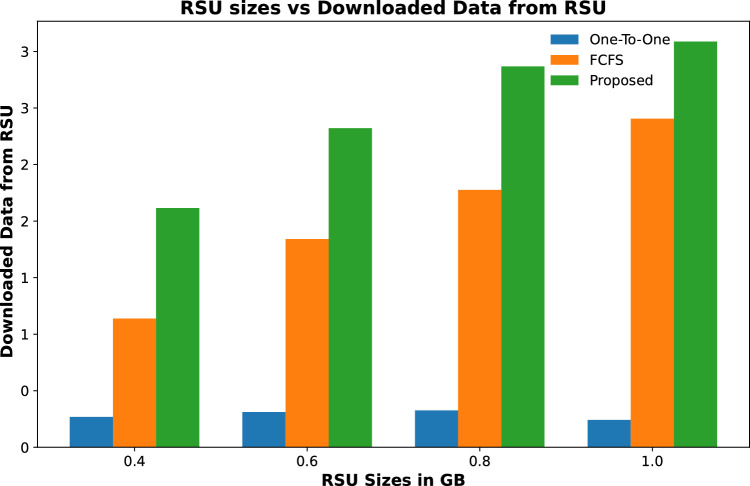
Data downloaded from contents cached on RSU against varying caching capacity of RSUs.

Downloaded data of all contents that are cached on fog-enabled RSUs are calculated in this section. Results in Figs. 12 and 13 show that the total downloaded data of contents cached from RSUs in the proposed scheme is more than the other two schemes. This is due to the maximum number of popular contents being cached on RSUs.

Figure 12 shows that for increasing the number of RSU, the downloaded data from the cached contents in the proposed scheme is increased from 8 GBs to 28 GBs for 2 to 5 RSUs respectively. However, the other two schemes download less amount of data from the cached contents. The same trend is shown in Fig. 13, when the cached size of fog nodes is increased for a fixed number of RSUs, that is 3. The results show that the proposed scheme downloads 24GB of data when the cache capacity of each RSU is 4 GB. However, the other two schemes could only download 16 GB of data.

**Figure 14:**
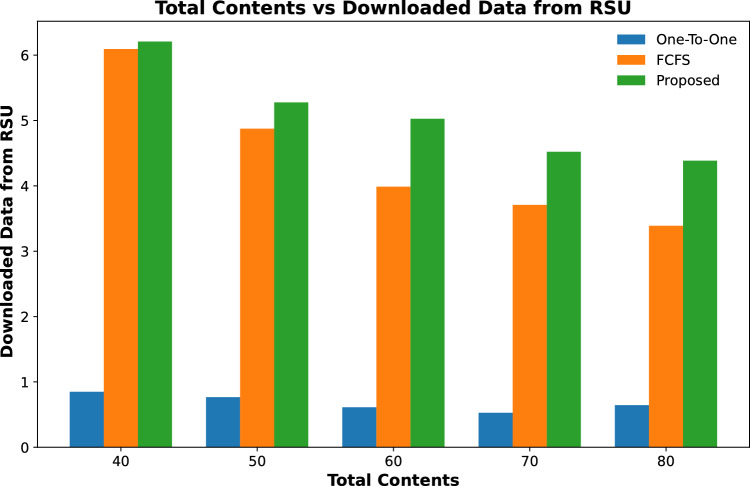
Data downloaded from contents cached on RSU against varying number of contents request.

The results shown in Fig. 14 are observed to download the data from the cached contents for varying numbers of content requests received from 100 vehicles. It is evident from the results that the proposed scheme downloads more data from the cached contents as compared to the other two schemes. The downloaded data from the cached contents decreases with the increase in number of contents. This is because the fog node is unable to cache all demanded contents on a fixed number of RSUs, resulting in less downloaded data from the same number of vehicles.

## Conclusion

This work proposes efficient content caching criteria for vehicles traveling in smart cities. The scheme proposes an algorithm for TCC to assign demanded contents on the cache by applying a modified Gale–Shapley stable matching algorithm. In addition, a utility function for both RSUs and contents is proposed to assign a preference list. The results are compared with a simple Gale–Shapley-based stable matching algorithm and FCFS. The results show that the proposed scheme caches 3% to 14% more popular content on RSUs from its close competitor. Similarly, the proposed scheme reduces average content downloading time from 13.33% to 52% and allows at least 33% to 50% more downloaded data from the contents cached on different RSUs as compared to one of the closely matched competitors.

## Data Availability

All related data is available in the paper.
